# Functional differences between the extraordinary eyes of deep-sea hyperiid amphipods

**DOI:** 10.1098/rspb.2024.0239

**Published:** 2024-05-29

**Authors:** Anna-Lee Jessop, Zahra M. Bagheri, Julian C. Partridge, Karen J. Osborn, Jan M. Hemmi

**Affiliations:** ^1^ School of Biological Sciences & Oceans Institute, The University of Western Australia, Perth, WA 6009, Australia; ^2^ Smithsonian National Museum of Natural History, Washington, DC 20560, USA; ^3^ Monterey Bay Aquarium Research Institute, Moss Landing, CA 95039, USA

**Keywords:** low light vision, spatial summation, midwater adaptations, retinal topography, visual field

## Abstract

The ocean's midwater is a uniquely challenging yet predictable and simple visual environment. The need to see without being seen in this dim, open habitat has led to extraordinary visual adaptations. To understand these adaptations, we compared the morphological and functional differences between the eyes of three hyperiid amphipods—*Hyperia galba*, *Streetsia challengeri* and *Phronima sedentaria*. Combining micro-CT data with computational modelling, we mapped visual field topography and predicted detection distances for visual targets viewed in different directions through mesopelagic depths. *Hyperia*'s eyes provide a wide visual field optimized for spatial vision over short distances, while *Phronima*'s and *Streetsia*'s eyes have the potential to achieve greater sensitivity and longer detection distances using spatial summation. These improvements come at the cost of smaller visual fields, but this loss is compensated for by a second pair of eyes in *Phronima* and by behaviour in *Streetsia*. The need to improve sensitivity while minimizing visible eye size to maintain crypsis has likely driven the evolution of hyperiid eye diversity. Our results provide an integrative look at how these elusive animals have adapted to the unique visual challenges of the mesopelagic.

## 1. Introduction

The unique visual environment within the ocean's expansive water column, or midwater, combined with the relentless selective pressures to see without being seen in an environment with nowhere to hide, have led to many adaptations. The study of midwater animal functional morphology offers a unique opportunity to gain insights into not only the evolutionary responses of organisms to pronounced and definable selective pressures, but also the characteristics of the Earth's largest, yet least explored, habitat.

Solar illumination varies in visually important ways from the ocean's surface to the bottom of the mesopelagic zone (1000 m), becoming less variable, dimmer, and spectrally restricted with depth [1]. These light conditions, combined with the many camouflage strategies common here [2] and the diverse bioluminescence emissions produced by deep-sea animals [3], have driven the development of extraordinary visual adaptations [4]. Perhaps the most notable of these adaptations are the disproportionally large eyes of some alciopid polychaetes, squids, fishes and hyperiid amphipods [4].

Hyperiid amphipods (Crustacea, Malacostraca, Peracarida) provide an ideal opportunity to investigate visual adaptations to the midwater. Hyperiids have a tractable evolutionary history [5] and, despite the group consisting of only approximately 240 described species, their eye diversity rivals even that of the insects [6,7]. Hyperiids' apposition compound eyes consist of tens to thousands of individual ommatidia, but ommatidial configurations differ between genera, species and even sexes [6,8–10]. What has driven the evolution of this diversity is unclear, but a few theories exist.

The leading hypothesis is that there is a correlation between the depth at which each species lives and its eye morphology. Land [6] suggested that hyperiids living at deeper depths exhibit greater dorsoventral asymmetry in their vision, hypothesizing that vision directed upward into downwelling light becomes more important at greater depths, while vision directed elsewhere is reduced. However, the available data on the depth ranges of hyperiids, primarily obtained from midwater trawls, lack the precision needed to accurately define their depth distributions. Given that the majority of hyperiids inhabit the upper 500 m and many exhibit diel vertical migrations, depth is almost certainly not the sole evolutionary driver of hyperiid visual system diversity.

We will argue here that host interactions and hyperiid swimming abilities are major contributors to the diversity of their eyes. Hyperiids are generally classified as parasites or commensals on gelatinous zooplankton [11,12]. The nature of these associations, as free-swimming predators or attached to the host, may change the visual tasks of hyperiids and thus may explain some of the differences between their eyes.

Determining the functional capabilities of hyperiid eyes is an important step towards understanding the evolutionary drivers of hyperiid eye diversity and offers a window into the selective pressures at work in the mesopelagic. Understanding the visual capabilities of different hyperiids also gives us clues to the behaviours of these difficult to observe animals’ and the important roles they play in midwater communities.

A first step in determining the visual capabilities of different compound eyes is to map the visual topography of different eyes. Recently a method based on micro-computed tomography (micro-CT) was developed to map the entire visual field of preserved compound eyes [13]. This method makes it possible to calculate the distribution of interommatidial angles and facet diameters across the entire visual field, providing a high-resolution reconstruction of each species' visual field. In this study, we combine this mapping method with a new computational modelling approach [14] to predict the functional capabilities of three very different hyperiid eyes (electronic supplementary material, figure S1). We mapped the dome-like eyes of *Hyperia galba* (Montagu, 1813), the two pairs of eyes in *Phronima sedentaria* (Forskäl, 1775), and the cylindrical eyes of *Streetsia challengeri* Stebbing, 1888 (electronic supplementary material, figure S1). We provide detailed descriptions of their visual fields, identify their visual specializations, and predict how two areas of their visual field perform on three target detection tasks. We also explore the effects of temporal and spatial summation on the functional capabilities of the three eye designs.

## 2. Methods

### (a) Specimens

*Hyperia galba* specimens (electronic supplementary material, table S1) were collected on December 2016 from the R/V *Western Flyer* using a modified midwater Tucker trawl with a 3 m^2^ opening, 500 µm mesh, and a bucket-style cod-end, deployed off Monterey, California, USA (36°42′18″ N, 122°3′18″ W) and towed obliquely from approximately 600 m depth to the surface. *Phronima sedentaria* specimens (electronic supplementary material, table S2) were collected on July 2018 from the R/V *Hugh R*. *Sharp* using a modified midwater Tucker trawl with a 2.25 m^2^ opening, 500 µm mesh and a closing cod-end, deployed off Lewes, Delaware, USA (37°42′15″ N, 73°37′21.8″ W) and towed obliquely from approximately 600 m depth to the surface. *Streetsia challengeri* specimens (electronic supplementary material, table S3) were collected on July 2017 from the R/V *Sonne* using a modified midwater Tucker trawl with a 45 m^2^ opening, 5 cm to 0.5 mm mesh, and a bucket-style cod-end, deployed approximately 700 km southeast of Sri Lanka (1°13′8.46″ N, 85°23′3.06″ E) and towed obliquely from 800 m depth to the surface.

Many widespread midwater species, including hyperiids, represent multiple species that have not yet been morphologically distinguished (e.g. [10,15,16]). For this reason, specimens are hereinafter referred to only by their genus names.

### (b) Sample preparation and micro-CT scanning

Specimens were euthanized by being immersed in iced seawater or the addition of a few drops of 95% ethanol to their holding tank. The heads and anterior-most segments of *Hyperia* and *Phronima* were dissected from the body, then fixed in 4% seawater-buffered formalin (*Hyperia*) or 2% cacodylate-buffered glutaraldehyde (*Phronima*). *Hyperia* and *Phronima* specimens were run through a dehydration series then stained with a 0.5% phosphotungstic acid (PTA) solution in 70% ethanol for 30 days before scanning. *Streetsia* specimens were fixed in 4% phosphate-buffered paraformaldehyde and stained with an aqueous 1% w/v iodine and 2% w/v potassium iodide solution for seven days. Specimens were mounted in 500 µl sealed Eppendorf tubes stabilized with either minute blocks of foam or 1% low-temperature-gelling agarose. *Hyperia* and *Phronima* specimens were scanned using a GE Phoenix V|tome|x M 240/180 kV Dual Tube micro-CT at the Smithsonian National Museum of Natural History at 80–90 kV and 2.65–4.26 W, with optical magnification of 70.8 and 36.6×. S*treetsia* specimens were scanned with a Versa 520 XRM (Zeiss, Pleasanton, CA, USA) at 70 kV and 6 W with 4× optical magnification at the University of Western Australia. Voxel sizes of 2.82–11.17 µm were achieved and the standard 0.7 kernel size reconstruction filter was used. Raw projection data from *Hyperia* and *Phronima* were reconstructed using datos|X (GE Sensing and Inspection Technologies) and visualized and exported using VG Studio (Volume Graphics), while XRM Reconstructor software (v. 10.7.3679.13921, Zeiss) following a standard centre shift and beam hardening (0.1) correction was used for *Streetsia*.

Average measurements of visual parameters were similar between both specimens of each genus (electronic supplementary material, tables S1–S3); thus specimen 1 of each genus is discussed unless otherwise noted. Damage, in the form of disconnected corneas and crystalline cones, depressions in the cornea, misalignment of rhabdoms and their crystalline cones, and aberrations of the retina, occurred in less than 5% of ommatidia in specimen 1 of each genus, and in less than 17% of ommatidia in specimen 2 of each genus. Damaged ommatidia, which were typically on the outer margins, were excluded from optical axes analyses but included in overall counts of ommatidia.

### (c) Analysis

#### (i) Optical axes and field of view

Three-dimensional optical axes of each ommatidium were mapped following the methods of Bagheri *et al.* [13]. The optical axes of ommatidia were then used to reconstruct the field of view of each specimen and each optical axis mapped onto a surrounding sphere [13]. The raw data were smoothed using an averaging algorithm in which the viewing direction of each ommatidium was replaced with the average viewing directions of its six neighbours over five iterations. The difference in calculated mean interommatidial angles between smoothing over one iteration or five iterations was less than 2% for *Hyperia* and *Phronima* and less than 4% for *Streetsia*.

#### (ii) Distribution of interommatidial angles

In *Hyperia* and *Phronima,* interommatidial angles (Δ*ϕ*) were calculated by averaging the angles between the optical axis of the selected ommatidium and its six neighbouring ommatidia (electronic supplementary material, figure S2A). Horizontally (anterior/posterior) the eye of *Streetsia* is almost four times longer than vertically (dorsal/ventral) with ommatidia arranged as a hexagonal array where rows of ommatidia are aligned to wrap around the head in vertical planes (electronic supplementary material, figure S3B). Therefore, for the *Streetsia* specimens, we calculated interommatidial angles separately in the vertical and horizontal directions, following previous methods [17]. We determined the horizontal interommatidial angle (Δ*ϕ*_h_) by calculating the angle between the optical axes of vertical rows of ommatidia. The vertical interommatidial angle (Δ*ϕ*_v_) was calculated as half the angle between the optical axes of neighbouring ommatidia along each vertical row (electronic supplementary material, figure S2B).

#### (iii) Facet diameters and acceptance angles

The average distance between the corneal point of each ommatidium and its six adjacent neighbouring ommatidia was used to estimate the facet diameter of ommatidia [13]. Using pseudopupil measurements, Nilsson [9] estimated an acceptance angle to interommatidial angle ratio (Δ*ρ*/Δ*ϕ*) of approximately 1.5 for *Hyperia*. We used this ratio to calculate the acceptance angle based on the interommatidial angles we measured for *Hyperia*.

For *Phronima*, acceptance angles were estimated as the rhabdom width (*d*) divided by the focal length (*f*) of the crystalline cones [18]. The focal lengths of individual ommatidia were assumed to be the lengths of the crystalline cones [8], measured as the distance between the points marked on the cornea and the distal tips of the light guides. Focal lengths and rhabdom widths of 50 random ommatidia from each eye of *Phronima* were measured using Dragonfly (Object Research Systems) and their averages were used to calculate the mean ommatidial acceptance angle.

For *Streetsia*, Land [6] measured an asymmetrical pseudopupil that extended across 20 × 1 ommatidia (horizontal × vertical) in the dorsal part of the eye and 5 × 1 in the lateral parts of the eye. Applying these ratios to our measured horizontal and vertical interommatidial angles, provides acceptance angle estimates that are larger in the horizontal than in the vertical direction (8° versus 2.1° and 6.5° versus 5.2° respectively). For simplicity, we assumed symmetrical receptive fields and estimated acceptance angles based on horizontal measurements.

### (d) Modelling the maximum detection distances

To compare the visual detection abilities between each genus we used the computational models developed by Bagheri *et al*. [14] for apposition compound eyes, which are based on models originally developed for camera-type eyes by Nilsson *et al*. [19]. In brief, the models allow us to estimate the maximum detection distances of visual targets such as luminescent point sources and extended dark objects that are seen against background radiance that varies with depth and viewing direction. The models take a statistical approach to visual detection in which the discrimination of a target against a background depends on the difference in photon counts between two channels: a target channel directed at the target and a background channel directed at the light field adjacent to the target within the animal's visual field. Discrimination between signals is only possible when the difference between the two channels is greater than or equal to a reliability coefficient *R* multiplied by the standard deviation of the difference, which is the square root of the sum of the two means [18].

We estimated the maximum detection distance of a point source (assuming an emittance of *E* = 10^10^ photons s^−1^ in accordance with previously reported flash intensities of bioluminescent organisms; reviewed in [20]) and a 1 cm diameter dark object with 50% transparency (a typical size of semi-transparent hyperiid prey). We modelled each target for upwards (against downwelling radiance) and lateral (against horizontal radiance) viewing directions through mesopelagic depths ranging from 200 to 600 m.

It should be noted that while we used the models of Bagheri *et al.* [14], we noticed that in that paper, for modelling point sources, but not the other targets, we had modelled the acceptance angle as one standard deviation of a Gaussian receptive field, rather than the full width at half height of the receptive field. This leads to an overestimation of detection distances, which has been corrected in all modelling here.

Each channel was modelled either as a single ommatidium or as a pool of neighbouring ommatidia in which photon catch is summated over an integration time of Δ*t* = 0.037 s [21]. Since *Streetsia* has an asymmetrical pseudopupil, we assumed that the pool of summating ommatidia in this genus constitutes a linear array of ommatidia within a horizontal column (electronic supplementary material, figure S3B). In *Hyperia* and *Phronima* the pseudopupils are more or less symmetrical; therefore we assumed the pools of ommatidia form hexagonal arrays in these genera (electronic supplementary material, figure S3A). Model parameters are shown in electronic supplementary material, tables S1–S4.

### (e) Depth ranges

Video observations recorded by the Monterey Bay Aquarium Research Institute's (MBARI's) remotely operated vehicles (ROVs) *Ventana, Tiburon* and *Doc Ricketts* were reviewed after retrieval from MBARI's Video Annotation Reference System (VARS; [22]), which contains all identified video observations of the target genera made with MBARI's ROVs since 1988. Identifications of *Hyperia*, *Phronima* and *Streetsia* in video observations were possible only to genus level. The ‘transit’ search resolution of the ROV video does not allow the identification of animals less than 1 cm in size, explaining the low observation counts for *Hyperia* and smaller *Phronima* and *Streetsia*.

## 3. Results

### (a) Depth ranges

Analysis of 762 video observations revealed similar depth ranges for *Hyperia* (31 observations), *Streetsia* (166 observations) and *Phronima* (565 observations; electronic supplementary material, figure S4). Ninety per cent of *Hyperia* observations were between 200 and 275 m, *Phronima* between 90 and 390 m, and *Streetsia* between 200 and 300 m depth.

### (b) Visual topography

**Figure 1.**
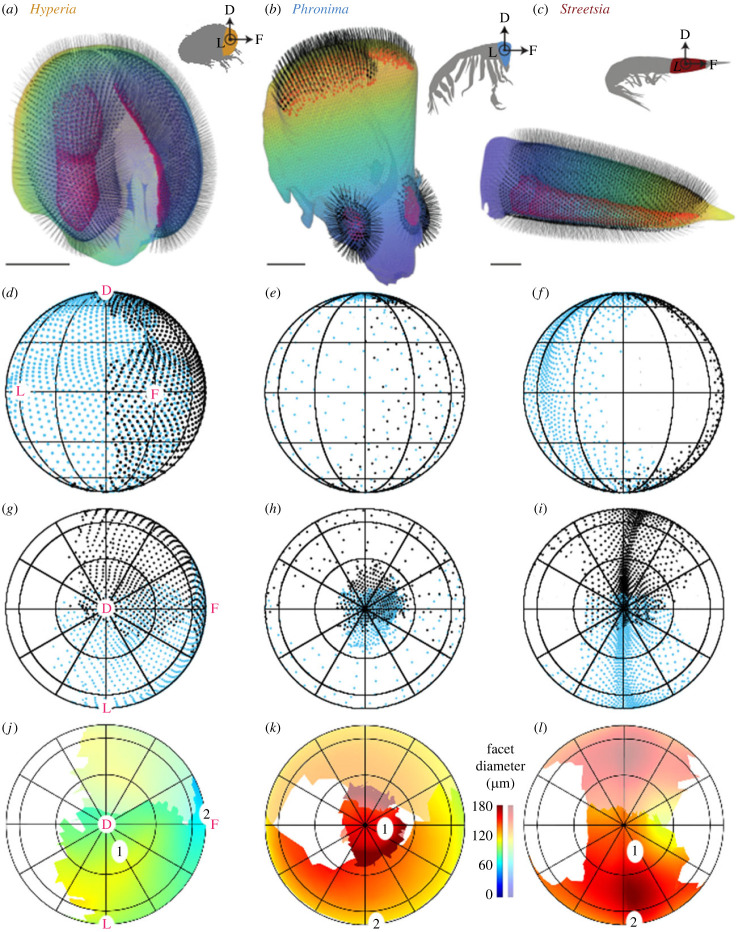
Eye shape, ommatidial viewing directions and distribution of facet diameters differed between *Hyperia* (left column), *Phronima* (centre column) and *Streetsia* (right column). (*a–c*) Ommatidial axes (black lines), defined by connecting centres of corneas (black dots) to centres of rhabdom/light guides (red dots), are shown on eye shapes of specimen 1 of each genus. Arrows and pink letters indicate the orientations of the visual fields: D, dorsal; L, lateral; F, frontal. Scale bars 1 mm. (*d–i*) Ommatidial viewing directions mapped from the centre onto a surrounding sphere (blue: right eye, black: left eye) in oblique and dorsal view. (*j–l*) Facet diameters (µm) across the dorsal visual field indicating relative sensitivity. Right eyes are shown in saturated and left eyes in unsaturated colours. Numbers on maps indicate regions from which visual parameters were taken for modelling dorsal (1) and frontal/lateral (2) regions.

The overall structures of the visual fields were consistent within genera (figure 1; electronic supplementary material, figures S5–S7) despite small differences between specimens, such as the total number of ommatidia and overall specimen size (electronic supplementary material, tables S1–S3).

The ommatidial axes of *Hyperia* were relatively uniformly distributed across the visual field, without obvious regional specializations (figure 1; electronic supplementary material, figure S5). The vertical and horizontal visual fields of each eye were approximately 200–270° and 130–140° respectively, with approximately 60 and 20° of binocular overlap frontally and dorsally respectively. This provides a combined visual field of approximately 270° vertically and 210° horizontally (figure 1; electronic supplementary material, figure S5), leaving a large, approximately 90° × 150° (V × H) posterior blind spot (figure 1*g*).

The optical axes of *Phronima*'s medial eyes all pointed dorsally (figure 1). Each eye had a relatively uniform but narrow (35° diameter) field of view providing a combined field of view of approximately 50° diameter, with 20° of binocular overlap (figure 1*h*). The optical axes of the lateral eyes covered a much broader field of view, with each eye covering over 180° vertically and 210° horizontally with approximately 60° of frontal overlap (figure 1). *Phronima* had only an approximately 30° diameter blind spot in the dorso-posterior part of the visual field (figure 1*h*).

The ommatidial axes of *Streetsia* were not uniformly distributed. Most axes converged on a prominent vertical visual streak that extended from dorsal to ventral across the full lateral visual field (figure 1). The dorsoventral visual field of each eye extended just over 220° (figure 1). This resulted in a 360° vertical visual field with approximately 20° and 60° of binocular overlap dorsally and ventrally respectively. Horizontally, each eye has a visual field of about 110–130°, resulting in blind spots (approx. 60° wide) in both the front and the back (figure 1).

### (c) Optical parameters

#### (i) Hyperia

**Figure 2.**
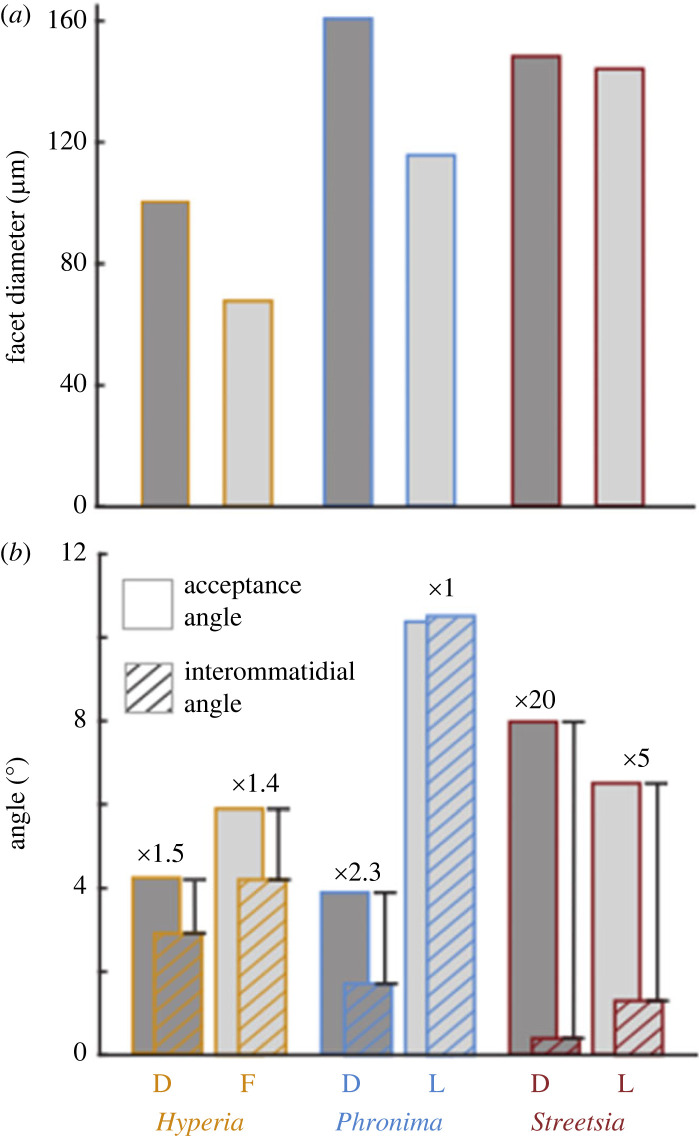
Facet diameter (*a*) and acceptance and interommatidial angles (*b*) from specimen 1 used as inputs for the modelling of detection distances (figure 3; electronic supplementary material, figure S8) from the dorsal (D, region 1 in figure 1) and frontal/lateral visual fields (F/L, region 2 in figure 1). Numbers above bars indicate the ratio of acceptance to interommatidial angle (*Hyperia*: [9]; *Phronima*: calculated; *Streetsia* horizontal: [6]).

The dorsal part of *Hyperia's* visual field had the highest sampling density (smallest interommatidial angles) and the largest facets (region 1; figures 1 and 2; electronic supplementary material, figure S6 and table S1). The frontal visual field also had relatively small interommatidial angles but had smaller facets (region 2; figures 1 and 2; electronic supplementary material, figure S6 and table S1). The ventral visual field had the lowest sampling density and smallest facets (electronic supplementary material, figure S6). Interommatidial angles were approximately 1.5 times smaller in the dorsal (2.9°) compared with the frontal region (4.2°; figure 2; electronic supplementary material, figure S6 and table S1). The opposite was true for facet diameters, which were 1.5 times larger in the dorsal (100.8 µm) compared with the frontal region (67.9 µm; figures 1 and 2; electronic supplementary material, figure S6 and table S1). Average crystalline cone length was substantially longer in the dorsal (891.3 µm) than the frontal (522.1 µm) region (electronic supplementary material, table S1). The average rhabdom width did not vary between frontal and dorsal eye regions (electronic supplementary material, table S1). Acceptance angles were slightly smaller in the dorsal than the frontal region (4.3° and 5.9° respectively; electronic supplementary material, table S1).

#### (ii) Phronima

*Phronima*'s medial and lateral eyes differed substantially in their distribution of sampling density (interommatidial angles) and facet diameters, with minimal overlap in their fields of view (figures 1 and 2; electronic supplementary material, figure S6). The average interommatidial angles in the medial eyes/dorsal region (1.7°) were approximately six times smaller than those in the lateral region (10.5°), while the average dorsal facet diameters were 1.5 times larger than the lateral facets (161 versus 115.6 µm respectively; figures 1 and 2; electronic supplementary material, figure S6 and table S2). Similarly, crystalline cones were longer in the dorsal (315.3 µm) than the lateral region (238.6 µm; electronic supplementary material, table S2). Rhabdoms were approximately two times narrower in the dorsal (21.4 µm) than the lateral region (43.3 µm; electronic supplementary material, table S2). These differences resulted in smaller estimated acceptance angles in the dorsal (3.9°) compared with the lateral region (10.4°), leading to a ratio of acceptance to interommatidial angles (Δ*ρ*/Δ*ϕ*) of 2.3 and 1, respectively (figure 2).

#### (iii) Streetsia

For *Streetsia*, sampling density and other parameters varied little between dorsal and lateral visual fields (figures 1 and 2; electronic supplementary material, figure S6 and table S3). There was a broad band of larger facets (148.3 µm) with smaller, horizontal interommatidial angles (0.4°) encircling the long axis of the animal in the middle of the eye (figure 1; electronic supplementary material, figure S6). These facets made up most of the eye and formed a pronounced vertical streak of high sampling density in the animal's visual field with a horizontal width of approximately 10°. Smaller facets (approx. 120 µm) were found on the ventral, anterior and posterior margins of the visual field (figure 1; electronic supplementary material, figure S6) and provided peripheral vision with low sensitivity.

Because of the elongated shape of *Streetsia*'s eyes (figure 1*c*), the vertical and horizontal interommatidial angles differed substantially. Vertical interommatidial angles (Δ*ϕ*_v_; electronic supplementary material, figure S7) were approximately seven and five times larger than horizontal interommatidial angles in the dorsal and lateral regions, respectively (electronic supplementary material, figure S7). Average crystalline cones were nearly four times longer in the dorsal than the lateral regions (1220.0 versus 330.1 µm, respectively; electronic supplementary material, table S3). The average rhabdom width did not vary between the dorsal and lateral region (electronic supplementary material, table S3). Approximated acceptance angles were consistently smaller in the dorsal visual field (figure 2; electronic supplementary material, table S3).

### (d) Visual performance

**Figure 3.**
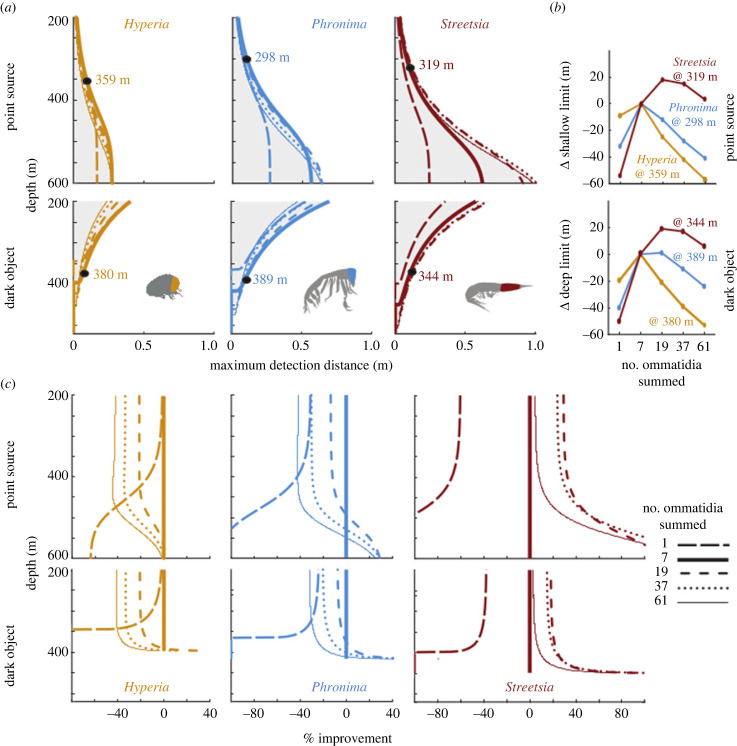
Detection distances and depth range of vision are increased with spatial summation for all targets. All values are compared with the performance when spatially summing seven ommatidia. (*a*) Detection distances are shown viewing against downwelling radiance across depths for the dorsal eye regions of *Hyperia* (left column), *Phronima* (middle column) and *Streetsia* (right column). Model results are shown for a point source (top row) and a 1 cm extended dark object with 50% transparency (second row). Grey shading indicates where detection can occur. Black dots indicate 10 cm detection distances used in (*b*). (*b*) Change in the upper and lower depth range of vision when detecting a point source (upper panel) and a 1 cm dark object (lower panel) viewed from 10 cm distance against downwelling radiance with increasing summation. (*c*) Percentage improvement for different amounts of spatial summation in the dorsal eye regions compared with the summation of direct ommatidial neighbours (7 ommatidia summed).

In all genera, luminous targets were seen at longer distances when viewed against the dimmer backgrounds encountered at greater depths or when targets were viewed against horizontal radiance (figure 3; electronic supplementary material, figure S8). As expected, dark objects were conversely detected at longer distances when viewed against the brighter backgrounds found in shallower depths (figure 3; electronic supplementary material, figure S8).

For point sources viewed from below, *Hyperia* had consistently shorter detection distances than *Streetsia* and *Phronima* (figure 3; electronic supplementary material, figure S8). Without spatial summation and even at 500 m depth, *Hyperia* was only able to detect point sources when they were within approximately 20 cm (electronic supplementary material, figure S8A). Under the same conditions, *Phronima* saw furthest (28 cm) and *Streetsia* just below 26 cm. If, however, as suggested by the overlapping fields of view of neighbouring ommatidia in their dorsal eyes, all genera employ spatial summation, these differences become substantially larger (electronic supplementary material, figure S8B). The benefit of spatial summation is most pronounced in *Streetsia*. However, detection distances more than doubled for both *Phronima* and *Streetsia* when using summation at depths below 450 m during the day*. Hyperia* has the least amount of improvement from spatial summation because they have the least ommatidial overlap of the three examined genera (figure 3).

For dark objects viewed from below without spatial summation, *Hyperia* was unable to detect objects below a depth of 350 m (electronic supplementary material, figure S8G). *Phronima* and *Streetsia* could see dark objects down to nearly 370 and 400 m depth, respectively (electronic supplementary material, figure S8G). With spatial summation, there was again a large improvement in detection distances for *Streetsia* but not as much in *Phronima* and *Hyperia,* except at the deep edge of their visual range (figure 3).

### (e) Spatial and temporal summation

Spatial summation improved detection distances in all genera (figure 3*a,c*). *Hyperia* and *Phronima* see furthest when summing the seven directly neighbouring ommatidia (figure 3*a,c*). *Streetsia,* however, sees furthest when summing 19 neighbouring ommatidia (figure 3*a,c*). Further summation beyond 7 and 19, respectively, is detrimental for all targets and genera at their most frequently observed depth ranges (figure 3*c*; electronic supplementary material, figure S4).

Spatial summation improves not only detection distances*,* but also the functional depth range of vision, which we define here as the ability to detect targets that are 10 cm away. The ability to detect point sources against downwelling radiance becomes possible 10 m shallower for *Hyperia*, over 30 m shallower for *Phronima,* and 70 m shallower for *Streetsia* (figure 3*b*) when optimal spatial summation is employed (7, 7 and 19 ommatidia, respectively), compared with using a single ommatidium. The ability to detect a dark object became possible at 20, 40 and 70 m deeper for *Hyperia*, *Phronima* and *Streetsia*, respectively (figure 3*b*), with spatial summation than without. The same patterns were seen to result from spatial summation for horizontal viewing directions of point sources and dark objects, but at shallower and deeper depths, respectively (electronic supplementary material, figure S8).

Instead of spatially summating, the animals could also improve detection distances through temporal summation, but this would slow vision. Here, we have assumed an integration time that is typical of mesopelagic crustaceans (0.037 s; [21]). To compare spatial and temporal summation effects, we modelled multiples of this integration time to approximately match the improvements in the detection of point sources through optimal spatial summation (electronic supplementary material, figure S9). *Hyperia* would have to slow its vision down approximately twofold (0.074 s), *Phronima* fourfold (0.148 s), and *Streetsia* almost 19-fold (0.703 s; dashed lines in electronic supplementary material, figure S9).

## 4. Discussion

### (a) Depth and eye design

*Hyperia, Phronima* and *Streetsia* all have large eyes and some form of dorsoventral asymmetry, which is hypothetically an adaptation to life in the deep sea [6]. This asymmetry is much stronger in *Phronima* than in *Streetsia* or *Hyperia* (figure 1), but there is no significant difference in their respective depth distributions based on direct observations gathered over a 32 year period off the coast of California (electronic supplementary material, figure S4). While depth and the accompanying differences in the light environment most certainly play a role in hyperiid eye evolution, most likely in the form of larger acceptance angles for deeper living species [14] and by limiting the effective range over which different eye designs can see (figure 3), these factors do not provide a convincing explanation of hyperiid eye diversity in epi- and mesopelagic waters. By contrast, we argue that eye morphology can be linked to behaviour, particularly their locomotory abilities, and their interactions with other animals. It should also be emphasized that we modelled the light field with maximum surface irradiance (midday sun) and assumed clear oceanic water (Jerlov Type 1; Jerlov [23]). This means our results are likely overestimates for dark object detection and underestimates for luminous objects. The light conditions of the greatest daytime depths we modelled are, at night, present all the way up to the surface [24].

### (b) *Hyperia*'s visual system

*Hyperia*'s pair of large dome-shaped eyes provide a broad visual field that spans over half the visual sphere in a relatively uniform way (figure 1*d,g*). *Hyperia*'s vision is primarily directed frontally and dorsally (figure 1; electronic supplementary material, figures S5 and S6), with some binocular overlap and only a slight asymmetry between dorsal and frontal/lateral vision compared with *Phronima* and *Streetsia*.

Our estimates of interommatidial angles (2.3–12.4°; electronic supplementary material, figure S6) were similar to the range reported by Nilsson [9] (0.8–10.5°). Nilsson's measurements were based on four types of ommatidia from different areas of the eye—one dorsal, two central and one ventral. We estimated angles across the entire eye, but found essentially the same range, though slightly larger values at either end of the range. These similarities suggest our micro-CT-based method is consistent with Nilsson's methods for determining interommatidial angles.

Spatial summation can be beneficial for improving detection distances when neighbouring ommatidia have overlapping receptive fields [8,14]. *Hyperia* has approximately equal interommatidial and acceptance angles [9]*,* meaning there is little overlap between the receptive fields of neighbouring ommatidia. However, even with such a low interommatidial to acceptance angle ratio (figure 2), our model still predicted an improvement of *Hyperia*'s vision from spatially summating directly neighbouring ommatidia (figure 3). There is no further benefit in depth range of vision (figure 3*b*) nor in the distance of detection (figure 3*c*), though, when summating across more distant ommatidia. Among the three genera, *Hyperia* has close to optically ‘optimal sampling (Δ*ρ*/Δ*ϕ* ≈ 1)’, which is typical for imaging systems optimized to maximize spatial resolution of a receptor matrix [25]. It also has a wide enough visual field and high enough sampling density for spatial vision (the ability to see shapes). This spatial vision would be compromised by spatial summation.

Temporal summation would be an alternative way to increase detection distances (electronic supplementary material, figure S9), but at the cost of slowing vision down, making it a poor option for fast-moving animals or for seeing fast-moving objects [26]. *Hyperia*, however, are primarily found living as ectoparasites on medusae [12,27], where they feed and deposit their young [28]. Observations in the ocean and in the laboratory suggest they spend little time free-swimming compared with *Phronima* and *Streetsia* (KJ Osborn, unpublished observation). This also means that they have limited control over their depth and, together with their hosts, may spend more time in the well lit epipelagic water (0–200 m), providing another possible reason why *Hyperia* has not evolved more specialized eyes.

In addition, visual predators in the open ocean are most likely to attack from below or horizontally [19], where there would not be sufficient contrast against the dark background for *Hyperia* to see them even 10 cm away (electronic supplementary material, figure S8). This makes it unlikely *Hyperia* uses vision to avoid predators. Furthermore, *Hyperia* may not need to detect predators because it avoids them behaviourally by living in close association with stinging, gelatinous animals that are not a primary target for most midwater visual predators [29,30]. High temporal resolution may therefore be less important in the evolution of *Hyperia*'s visual capabilities, allowing temporal summation (electronic supplementary material, figure S9).

The depth range within which *Hyperia* lives is poorly known and our video records consist of only 31 ROV observations. It is assumed they spend the bulk of their time in the top 200 m of the water column (e.g. [31,32]), though reliable reports down to 1100 and 2000 m also exist [33,34]. Further complicating the picture, *Hyperia* vertically migrates [35] and the depth range of effective vision changes with solar illumination and water clarity. A better understanding of the depth range of *Hyperia* would clarify the conditions their vision operates in.

In summary, *Hyperia*'s habit of living in close association with their host medusae reduces their need to detect predators or prey at great distances. As a result, they have large eyes with large, dark retinas that are easily visible, and eyes that are well suited for spatial vision over short distances. *Hyperia* may benefit from temporal summation. This is consistent with their sedentary life interacting with conspecifics on a medusa. We can therefore predict that *Hyperia* has slower eyes than the other two genera.

### (c) *Phronima*'s visual system

*Phronima* is famous for the extreme asymmetry between its two eye pairs [8,36]. The smaller, lateral eyes have large visual fields reminiscent of those of the more dome-shaped eyes of *Hyperia,* but *Phronima*'s lateral eyes have far fewer ommatidia and lower sampling densities (figure 1; electronic supplementary material, figures S5 and S6 and tables S1 and S2). Together, *Phronima*'s lateral eyes cover almost the entire visual sphere (figure 1; electronic supplementary material, figure S5), giving *Phronima* the largest visual field of the three genera examined here. This large field of view is the result of ommatidia with large acceptance angles and little overlap between their receptive fields. These lateral eyes, however, have much shorter detection distances for all visual targets compared with their medial eyes (electronic supplementary material, figure S8; [14]).

Extreme dorsal/ventral ocular asymmetry is also found in several mesopelagic fishes and the cockeyed squid, *Histioteuthis heteropsis*. However, based on anatomical approximations the angular extent of their visual fields is more than twice that of *Phronima's* combined medial eyes [4,37,38]. It has been suggested that the angular size of the visual field may be related to the three-dimensional radiance distribution of downwelling light [6]. Indeed, in some midwater animals such as the *Histioteuthis*, the dorsal visual field is thought to correspond closely to the angular size of Snell's window (97°). At greater depths the downwelling light is brightest directly above the viewer and it decreases to half this level at approximately 35° either side of the vertical, but the angular size of this downwelling light [1] is still significantly wider than the combined visual field of *Phronima*'s medial eyes (50°). *Phromina* lives in a gelatinous ‘barrel’, which we previously argued restricts *Phronima's* visual field to the narrow opening of the barrel and therefore lessens the costs of reducing visual field size to improve spatial summation [14]. Alternatively, the presence of a second pair of eyes with a visual field that complements the medial eyes may have the same effect. As the medial visual field decreases, the lateral eyes cover the lost field of view (figure 1; electronic supplementary material, figure S6), although with a reduced functional depth range and shorter detection distances (electronic supplementary material, figure S8).

Our estimates of interommatidial angles in *Phronima*'s lateral eye (9–12°; electronic supplementary material, table S2), agrees well with Land [8]. But his estimates for the medial eyes (0.44–0.88°) differ from ours (0.98°–2.97°). Since we were able to measure interommatidial angles across the entire eye, differences in location of measurements are unlikely to explain these differences. Instead, the differences between individuals, sexes and species are a more likely explanation [10]. Further work is needed to document variation between individuals and sexes and to resolve species complexes so that observed differences can be correctly attributed.

The medial eyes have extremely narrow visual fields (approx. 35°) with high binocular overlap (approx. 20°) and their ommatidia have large facets with highly overlapping receptive fields. Without considering spatial or temporal summation, *Phronima's* medial eyes achieve the largest detection distances of all genera and targets investigated here (figure 3; electronic supplementary material, figure S8). Spatial summation further increases detection distances for both the medial and lateral eyes (electronic supplementary material, figure S8). The depth range of useful vision also broadens with spatial summation, as observed for all genera (figure 3; electronic supplementary material, figure S8). Temporal summation would increase detection distances, but a fourfold reduction in temporal resolution would be needed to achieve the same detection distances that spatial summation provides (electronic supplementary material, figure S9). Given *Phronima's* free-swimming behaviour, such a reduction in temporal resolution is unlikely to be adaptive.

### (d) Transparency as a predictor of eye anatomy

Another contributor to the narrow field of view of *Phronima*'s dorsal eyes may be the visibility of the retina to visual predators. Retinas are inevitably dark. Dark pigmentation is useful camouflage at depths where bioluminescent light is more important than solar illumination because it reduces reflection of bioluminescent wavelengths [39,40]. At shallower depths, in contrast, transparency is a more useful camouflage strategy [38]. Mostly transparent *Phronima* minimizes the visibility of its retinas by condensing them to a small spot at the centre of each eye's curvature. However, to allow this and keep interommatidial angles small, the eye must be exceptionally large. In *Phronima*'s case the eye radius is at least 90% of the head length and twice the body depth. With such a large eye radius, the visual field must be very small to keep the overall eye size manageable. So, *Phronima*'s narrow visual field may be driven by the need to keep its retina as condensed as possible. By contrast, *Hyperia* clearly does not face the same selective pressure for its eyes to be transparent because its body is generally darkly pigmented and typically associated with hosts that are not prized prey items [30]. This release from the need to be transparent allows *Hyperia* to greatly expand the size of its retina and therefore its visual field, while keeping sampling resolution relatively high and eye size small. Consequently, its retina forms a large dark sheet that would be easily seen in a transparent animal. *Streetsia* has essentially taken a similar approach to *Phronima* by tightly packing its retina in one dimension into a small structure. The compact retina forms a long, thin cylinder that extends the length of the eye parallel to the long body axis. It is likely that the visibility of the retina to visual predators is a major contributor to the exceptional diversity of hyperiid eye designs.

### (e) *Streetsia*'s visual system

*Streetsia* has the largest eyes of the three genera examined and yet has the most restricted visual field. However, because its specialized visual streak allows spatial summation, the modelled detection distances in the lateral visual field are longer for *Streetsia* than for *Phronima* and *Hyperia* (figure 3; electronic supplementary material, figure S8).

*Streetisa*'s vision is dominated by a narrow 10° band that forms a complete ring around the longitudinal axis of the animal's elongate eyes and body (figure 1*f, i*). This band is made up of horizontal rows of ommatidia pointed in the same direction (figure 1*f, i*). Unlike typical visual streaks that are used to improve spatial resolution [13,41,42], the ommatidia in *Streetsia*'s streak have significant receptive field overlap, reminiscent of the medial eye of *Phronima*. This overlap suggests that the streak is being used to maximize sensitivity through spatial summation [8,14], rather than to maximize spatial resolution (figure 3; electronic supplementary material, figure S8).

The orientation of the visual streak is likely related to *Streetsia*'s mode of locomotion. *Streetsia* is most often observed free-swimming in the water column (KJ Osborn, unpublished observation). While *Streetsia* could employ either temporal or spatial summation to improve visual detection distances (figure 3; electronic supplementary material, figure S9), temporal summation would be a poor option for a fast swimmer because it slows vision (electronic supplementary material, figure S9; [26]). While spatial summation does not slow vision, the need for overlapping acceptance angles does reduce the visual field, unless acceptance angles become extremely large, which would reduce detection distances and counteract the benefits of spatial summation [14].

The spatial distribution of the zone of high sensitivity vision in *Streetsia* is unusual among midwater animals. While it is likely that other animals would benefit from high-sensitivity vision in multiple directions, lateral vision is substantially more challenging than upward vision because lateral radiance is two orders of magnitude less than downwelling radiance in the mesopelagic zone in daylight hours [1]. It is nearly impossible to develop an eye large enough to achieve high sensitivity in multiple directions, yet *Streetsia* has achieved this. As a streamlined, continuous swimmer, *Streetsia* has been able to compress most of its ommatidia into a vertical visual streak for spatial summation and therefore high sensitivity that it moves continuously through the environment like a line scanner. The extreme nature of this solution is highlighted by the fact that *Streetsia* has a large frontal blind spot (figure 1; electronic supplementary material, figures S5 and S6), which means the animal cannot see in the direction it swims. The elegance of *Streetsia*'s solution is that it has high-sensitivity vision surrounding their entire long body axis, not just upwards (figure 1; electronic supplementary material, figures S5 and S6).

Both *Streetsia* and *Phronima* have optically very different eyes compared with *Hyperia*. Both have eyes with large acceptance and small interommatidial angles resulting in significant receptive field overlaps that make spatial summation between adjacent ommatidia effective [8,14]. The use of spatial summation to increase sensitivity is, however, where the similarity ends because *Streetsia* and *Phronima* have evolved completely different strategies to deal with the resulting reduced visual field. *Phronima* has restricted its high-sensitivity vision to a small region of dorsally directed visual space (figure 1; electronic supplementary material, figures S5 and S6). From observations of *in situ* and laboratory animals, it seems unlikely that *Phronima* consistently points its medial eyes upward (KJ Osborn, unpublished observation; [43]). Being frequently inside a barrel, lacking a streamlined body and appendages, and having a different swimming motion from *Streetsia*, may have prevented *Phronima* from developing a visual streak. In its absence, *Phronima* has a second pair of eyes that delivers close-range, but almost omnidirectional, vision (figure 1). While the difference between *Phronima*'s two eye pairs is particularly extreme, we see similar, though less developed double eyes in other closely related hyperiids, such as *Primno* and *Phrosina*.

## 5. Conclusion

Our modelling revealed that hyperiid amphipods with apposition compound eyes only have access to visual information over relatively short distances, on the order of tens of centimetres. Despite the vastness of the deep sea, vision in these animals is clearly limited in range. This has predictive implications for predation, predator avoidance, inter- and intraspecific signalling, and deep-sea community structure.

We have also shown that depth range *per se* is unlikely to be the main driver of hyperiid eye diversification. While the scarcity of light at greater depth often leads to increases in eye sizes and acceptance angles, the way animals structure their visual fields more strongly reflects differences in their visual ecology [44], with locomotory behaviours and the need to be transparent being two main drivers of eye evolution.

## Data Availability

Micro-CT scan data and all code and models are available on Dryad: https://doi.org/10.5061/dryad.prr4xgxtd [45]. Supplementary material is available online [46].
